# 2,3-Dimethylpentane and 2-Methylhexane as a Test Mixture for Evaluating Highly Efficient Fractionating Columns*

**DOI:** 10.6028/jres.067A.002

**Published:** 1963-02-01

**Authors:** Edwin C. Kuehner

## Abstract

A test mixture consisting of 2,3-dimethylpentane and 2-methylhexane was prepared and its relative volatility determined by a fractional distillation method. This test mixture was compared, experimentally and theoretically, with another test mixture commonly used for evaluating highly efficient fractionating columns.

## 1. Introduction

The development of more highly efficient fractionating columns has resulted in a greater need of test mixtures with lower relative volatilities than the ones commonly used in evaluating stills. The choice of components for such a test mixture is further limited to those which form ideal solutions and differ considerably from one another in a specific physical property, such as refractive index, by which the composition of mixtures of the components may be determined. The 2,2,4-trimethylpentane and *n*-heptane combination, with a normal boiling point difference of 0.812 °C, is an excellent test mixture for evaluating fractionating columns of medium efficiency. The separation of these components becomes sufficiently complete with fractionating columns of greatly increased efficiency, so that the number of theoretical plates, calculated by the Fenske equation [1],^1^ becomes sensitive to small analytical errors.

Two isomers of heptane were selected for the components of a test mixture which might fulfill the requirements for evaluating very highly efficient fractionating columns. They are 2,3-dimethylpentane and 2-methylhexane, with normal boiling points [2] of 89.784 °C and 90.052 °C, respectively, a difference of 0.268 °C. The refractive index at 20 °C [2] of 2,3-dimethylpentane is 1.39196, and that of 2-methylhexane is 1.38485, a difference of 0.00711. This difference in refractive index is almost twice the difference in refractive index of 2,2,4-trimethylpentane and *n*-heptane.

## 2. Experimental Procedure

### 2.1. Apparatus

A random-packed still having a column 25 mm in diameter and 300 cm in height was used in this work. This vacuum-jacketed column was packed with chromel spirals (Helipak) and further insulated with aluminum-covered glass wool.

Also used was a precision-packed still similar to the Podbielniak Heligrid type, but having a packing made of precision wound platinum wire. This vacuum-jacketed column was 25 mm in diameter by 100 cm in height and further insulated with aluminum-covered glass wool.

A differential refractometer with a rotating cell block and vernier eye piece was used to determine mixture composition. A gas chromatograph, Perkin- Elmer Model 154, equipped with a hydrogen flame ionization detector and a capillary column with squalene substrate, was used for determining the presence of other isomers in each of the test mixture components.

### 2.2. Materials

The components for the *n*-heptane—2,2,4-trimethylpentane test mixture were obtained from Phillips Petroleum Company. They were distilled and redistilled in the 300-cm random-packed still until all traces of impurities detectable by analysis with the gas chromatograph and the differential refractometer were removed.

Of the two components of the 2,3-dimethylpentane—2-methylhexane test mixture, only the 2,3-dimethylpentane was obtainable in better than 90 mole percent purity from commercial sources. This material also contained about 5 mole percent of the second component in the test mixture, 2-methylhexane, which did not have to be removed. A distillation with the 300 cm still removed all of the other impurities which were detectable with the gas chromatograph.

Commercial grade isoheptane, obtainable from Phillips Petroleum Company, was the only commercial source of 2-methylhexane. This material contained both of the desired components of the test mixture, but in amounts of less than 20 mole percent of each. The presence of considerable amounts of close-boiling naphthenes in the commercial material was responsible for the difficulty in obtaining a test mixture from this material.

Several 4,000 ml charges of isoheptanes were distilled with the 300-cm random-packed still. With the aid of the gas chromatograph, the fractions having a high concentration of the desired two isomers were selected; these were combined and redistilled. Only the fractions from this second distillation, which contained less than 2 percent naphthenes by chromatographic analysis, were combined for an attempt to purify further by azeotropic distillation. An equal amount of triethylamine was used as the azeotrope former, which was then separated from each fraction by subsequent extractions with ice water and dilute mineral acid. The fractions for which their respective chromatograms showed only two peaks, representing the desired isomers, were combined and percolated through silica gel to remove any remaining trace of triethylamine. By repeating this distillation procedure, 700 ml of 2,3-dimethylpentane—2-methylhexane test mixture, containing about 70 mole percent of 2-methylhexane, was obtained from 10 gallons of commercial isoheptanes.

Triethylamine was found to be more effective in removing traces of naphthenes than some of the other azeotrope formers that were tried, but complete removal was unsuccessful when more than 2 percent of naphthenes was still present in the isoheptanes after the second distillation.

The quantity of 2,3-dimethylpentane—2-methylhexane test mixture obtained by azeotropic distillation was sufficient for efficiency tests and relative volatility determination with the 100 cm precision packed still, but was insufficient for use in the 300-cm still having a greater hold-up in the column. Fortunately, a liter of synthetically prepared 2-methylhexane was obtained from the Chemistry Department of the Ohio State University. Because this material was of very high purity, further distillation was not necessary.

### 2.3. Calibration of Differential Refractometer

The determination of the composition of the test mixtures by gas chromatography was not possible because neither of the mixtures would separate completely, and the resulting peak areas could not be calculated with sufficient accuracy. The determination of composition by refractive index measurements, more precisely accomplished with a differential refractometer, was found to be the most expedient method of analysis.

Since the change in refractive index with change in composition of a test mixture is not entirely a linear relationship, it was necessary to calibrate the differential refractometer reading against a series of known compositions of the constituents for both test mixtures. Standard samples were used for this purpose, and the best equation of the curve was calculated by the method of least squares. The results, expressed as the difference between the refractive index of the mixture and one of their constituents, are as follows:

For 2,2,4-trimethylpentane and *n*-heptane Δ=ri(2, 2, 4-trimethylpentane)—ri (mixture) =0.0030*x* + 0.0008*x*^2^ where ri is the refractive index and *x* is the mole fraction of *n*-heptane.

For 2,3-dimethylpentane and 2-methylhexane Δ=ri (mixture)—ri (2-methylhexane) = 0.0073*x*—0.0002*x*^2^ where *x* is the mole fraction of 2,3-dimethylhexane.

### 2.4. Relative Volatility Determination

The relative volatility of the 2,3-dimethylpentane—2-methylhexane test mixture was determined by the fractional distillation method [3] and compared with the value calculated from vapor pressure data. By this method, the relative volatility of an unknown test mixture is determined with a still for which the efficiency was previously determined with a test mixture of known relative volatility. For the test mixture of known relative volatility, 2,2,4-trimethylpentane—*n*-heptane with a value of 1.0240 [4] was used. The 100-cm still was chosen to avoid exceeding the recommended maximum separation of 2,2,4-dimethylpentane and *n*-heptane [5]. This 100-cm still was charged with 700 ml of the 2,2,4-trimethylpentane—*n*-heptane test mixture and preflooded. Twenty-four hours later samples were taken of the distillate and of the returning material entering the still pot. The differential refractometer and the calibration equation for this test mixture were used to determine the composition of the samples. The number of theoretical plates *n* given in table 1 was calculated by means of the Fenske equation [1]. The average of seven determinations was used later to calculate the relative volatility of the 2,3-dimethylpentane—2-methylhexane test mixture.

The same procedure was employed with 700 ml of the 2,3-dimethylpentane—2-methylhexane test mixture. The same still was used under as nearly the same operating conditions as possible, and samples were taken and analyzed in a similar manner. The relative volatility *α* of this test mixture was calculated by the Fenske equation rearranged into the following form: $\log\;\alpha =\frac{1}{n}\log\;\left(x/1-x\right)\left(1-y/y\right)$ where *x* and *y* are the mole fractions of 2,3-dimethylpentane in the samples taken from the distillate and material returning to the still pot, respectively. The average of the three determinations (table 2) is in good agreement with the value calculated from the vapor pressure data [2,6] of 2, 3-dimethyplentane and 2-methylhexane at the temperature corresponding to the mean value of their normal boiling points. The value thus obtained should not be regarded as an absolute value for the relative volatility for this test mixture, but as a sufficiently good approximation to show some of the merits of this test mixture from both experimental and theoretical considerations.

### 2.5. Efficiency Test Runs

Efficiency test runs were performed, using the 300-cm random-packed still, with both the 2,2,4-trimethylpentane—*n*-heptane and the 2,3-dimethylpentane—2-methylhexane test mixtures. For each run, the still vaporizer was charged with 1,800 ml of one of the mixtures and preflooded. While operating at total reflux, samples of 2 ml each were taken of the distillate and the returning material at 24-hr intervals for a period of 7 days. During a run the rate of vaporization was controlled by a thermistor-actuated control device [7]. The composition of the samples was determined with the differential refractometer which was calibrated with known compositions of the two test mixtures. The number of equivalent theoretical plates was calculated from the composition of the samples of the distillate and material returning to the vaporizer by means of the Fenske equation. The values 1.024 [4] for the relative volatility of 2,2,4-trimethylpentane—*n*-heptane test mixture and 1.0079 for the 2,3-dimethylpentane—2-methylhexane test mixture were used in these calculations.

A standard deviation, *σ*_ri_ of 16 scale divisions or 0.000013 refractive index units, was determined with both test mixtures with the differential refractometer. By substituting the calibration equations for each test mixture into the standard propagation of error formula, equations for the standard deviation in terms of mole fraction were derived. For the 2,2,4-trimethylpentane—*n*-heptane test mixture the equations were *σ_x_*=*σ*_ri_/(0.0030+0.0016*x*) and *σ_y_= σ*_ri_/(0.0030 + 0.0016*y*). For the 2,3-dimethylpentane—2-methylhexane test mixture the equations were *σ_x_= σ*_ri_/(0.0073 — 0.0004*x*) and *σ_y_= σ*_ri_*/*(0.0073 — 0.0004*y*). The following equation was used to calculate the standard deviation, *σ_n_*, in terms of the number of theoretical plates:

$$\sigma_{n}^{2}=\frac{1}{{\left(\ln\;\alpha \right)}^{2}}\left[{\left\{\frac{\sigma_{x}}{x\left(1-x\right)}\right\}}^{2}+{\left\{\frac{\sigma_{y}}{y\left(1-y\right)}\right\}}^{2}\right]$$

For both test mixtures, *x* and *y* are the mole fractions of the lower boiling constituents in samples taken from the top and bottom of the still respectively; *σ_x_* and *σ_y_* are the standard deviations of *x* and *y* respectively. The latter equation was derived from the Fenske equation and the standard propagation of error formula in which the correlation coefficient is zero.

Data on an efficiency run for each test mixture, including the standard deviations in terms of mole fraction and number of theoretical plates are given in table 3. These standard deviations were propagated entirely from analytical errors involved in reading the differential refractometer. Errors involved in taking boil-up rates and other errors peculiar to still operation, which are extremely difficult to determine, were entirely excluded in any of the calculations. The data given in table 3 should not be interpreted as an actual evaluation of the still at a definite boil-up rate, but as an indication of the effect an analytical error has on the calculated number of theoretical plates for the two test mixtures. For this reason, actual boil-up rates were not included in the data.

## 3. Discussion

As shown in table 3, a small error in reading the differential refractometer corresponds to a much greater error in the calculated number of theoretical plates when the 2,2,4-trimethylpentane—*n*-heptane test mixture is used for evaluating a highly efficient fractionating column than when the 2,3-dimethylpentane—2-methylhexane test mixture is used. This higher error occurs because the difference in boiling points of 2,2,4-trimethylpentane and *n*-heptane is large enough to result in such complete separation that the calculated number of theoretical plates is greatly affected by analytical errors. Also, the difference in their refractive indices is considerably less than that of 2,3-dimethylpentane and 2-methylhexane, resulting in a lower analytical precision.

In order to make a graphical comparison of the corresponding *σ_n_* due to analytical errors over a wide range of theoretical plates *n* for both test mixtures, a common basis or condition was necessary. It has been pointed out [5] that the effect of random analytical errors on *n* is least when the mole fraction of the material returning to the vaporizer *y* is equal to (1*−x*), where *x* is the mole fraction of the lower boiling constituent in the distillate. This is sufficiently correct only when the magnitudes of the probable analytical errors of *x* and *y* are approximately equal. With this optimum condition imposed, the Fenske equation can be written as

$$n=\frac{2\;\log\;\left(x/1-x\right)}{\log\;\alpha }$$

and the standard propagation of error formula as

$$\sigma_{n}=\frac{{\left(\sigma_{x}+\sigma_{\left(1-x\right)}\right)}^{\frac{1}{2}}}{x\left(1-x\right)\left(\ln\;\alpha \right)}$$

**Figure 1:**
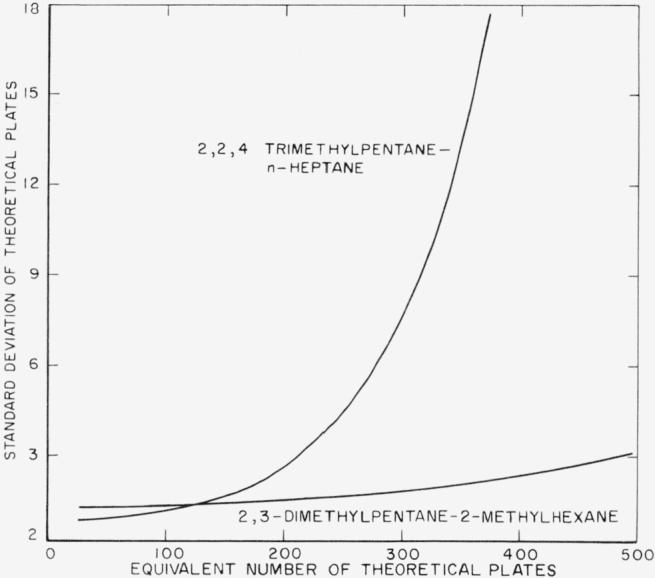
Variation of standard deviation in number of theoretical plates with number of theoretical plates Based on a standard deviation of 0.000013 refractive index units for both test mixtures and the condition that *y*=(1*−x*).

By calculating *x* for a series of *n* values and substituting these values into the propagation of error formula, a series of *σ_n_* values was obtained. The *σ_x_* and *σ*_(1_*_−x_*_)_ used in the calculation were obtained by the use of the error formula for the calibration of the differential refractometer and a standard deviation, (*σ*_ri_, of 16 scale division or 0.000013 refractive index units. The calculated *σ_n_* values are plotted against theoretical plates in figure 1 for both test mixtures. These curves show that, under the imposed condition, the 2,2,4-trimethylpentane—*n*-heptane test mixture is more desirable for evaluating stills developing less than 125 theoretical plates, but its desirability rapidly diminishes in evaluating stills greater than 125 plates. A still with 500 theoretical plates could be evaluated with the same standard deviation, *σ_n_*, when the 2,3-dimethylpentane—2-methylhexane test mixture is used as when a 225-plate still is evaluated with the 2,2,4-trimethylpentane—*n*-heptane test mixture.

Only the analytical errors in reading the differential refractometer were considered in determining the curves in figure 1; the other factors would have approximately the same effect on both test mixtures, when the stills are operated under the same conditions.

The 2,3-dimethylpentane—2-methylpentane test mixture has the advantage of having a very low relative volatility and a large difference in refractive index of its components which greatly extends its usefulness in evaluating more highly efficient stills. A disadvantage in using this test mixture is the increased cost involved in the preparation and purification of its components over other readily obtainable test mixtures. Since it is possible to use a given volume of test mixture repeatedly for evaluating a number of fractionating columns, this initial cost may not seriously hamper its desirability as a test mixture, especially in evaluating very highly efficient fractionating columns in which the separation of components of other test mixtures becomes too great for precise analysis.

## Figures and Tables

**Table 1:** Efficiency determination of 100 cm precision-packed still (2,2,4-trimethylpentane—*n*-heptane test mixture)

Determinations	Boil-up rate	Mole fraction		Number of theoretical plates
		Distillate	Bottom	
				
	*ml/min*			
1	20.7	0.6997	0.3508	60.6
2	21.2	.7052	.3483	62.2
3	21.0	.6468	.3483	61.3
4	20.2	.6981	.3642	57.8
5	20.4	.7271	.3556	65.4
6	20.8	.7056	.3513	61.7
7	20.2	.7176	.3642	61.8
Average	20.6	……	……	61.5

**Table 2:** Relative volatility determination of 2,3-dimethylpentane—2-methylhexane test mixture

Determinations	Boil-up rate	Mole fraction		Relative volatility
		Distillate	Bottom	
				
	*ml*/*min*			
1	20.3	0.5876	0.4681	1.00774
2	19.9	.5916	.4671	1.00807
3	19.5	.5880	.4660	1.00790
				
Average	19.9	……	……	1.00790
Value calculated from vapor pressure data [2,6]	……	……	……	1.00795

**Table 3:** Efficiency test runs on 300 cm random packed fractionation column

Test mixture	Days after pre-flooding	*x*	*σ_x_^a^*	*y*	*σ_y_*^a^	*n*	*σ_n_*
							
2,2,4 Trimethylpentane—							
*n*-heptane	1	0.9651	0.0029	0.1844	0.0040	202.7	3.2
	2	.9902	.0029	.1741	.0040	261.5	12.5
	3	.9938	.0029	.1690	.0040	281.2	19.6
	4	.9944	.0029	.1640	.0040	284.1	20.3
	5	.9942	.0029	.1584	.0041	287.3	21.0
	6	.9944	.0029	.1539	.0041	290.2	22.5
	7	.9944	.0029	.1490	.0041	291.9	22.5
2,3-Dimethylpentane—							
2-methylhexane	1	.6087	.0018	.2650	.0019	185.8	1.6
	2	.6760	.0018	.2587	.0019	227.2	1.6
	3	.6968	.0018	.2574	.0019	240.4	1.7
	4	.7006	.0018	.2584	.0019	242.0	1.7
	5	.7023	.0018	.2574	.0019	243.8	1.7
	6	.7196	.0018	.2538	.0019	256.8	1.7
	7	.7204	.0018	.2528	.0019	258.0	1.7

a Based on a *σ_ri_* of 0.000013 refractive index units.

## Footnotes

1 Figures in brackets indicate the literature references at the end of this paper
